# Terahertz imaging demonstrates its diagnostic potential and reveals a relationship between cutaneous dehydration and neuropathy for diabetic foot syndrome patients

**DOI:** 10.1038/s41598-022-06996-w

**Published:** 2022-02-24

**Authors:** Goretti G. Hernandez-Cardoso, Lauro F. Amador-Medina, Gerardo Gutierrez-Torres, Edgar S. Reyes-Reyes, César Augusto Benavides Martínez, Cuitlahuac Cardona Espinoza, José Arce Cruz, Irving Salas-Gutierrez, Blanca O. Murillo-Ortíz, Enrique Castro-Camus

**Affiliations:** 1 Centro de Investigaciones en Optica, A. C., Loma del Bosque 115, Lomas del Campestre, 37150 Leon, Guanajuato Mexico; 2 Department of Physics, Philipps-Universität Marburg, Renthof 5, 35032 Marburg, Germany; 3 UMAE No. 1 Bajio, Instituto Mexicano del Seguro Social, Leon, Guanajuato Mexico; 4 Hospital General de subzona #7, Instituto Mexicano del Seguro Social, San Francisco del Rincon, Guanajuato Mexico; 5 Unidad de Investigación en Epidemiologia Clinica, OOAD Guanajuato, Instituto Mexicano del Seguro Social, Leon, Guanajuato Mexico; 6 Hospital Angeles Leon, Av. Cerro Gordo 311, Lomas del Campestre, 37150 Leon, Guanajuato Mexico

**Keywords:** Terahertz optics, Biological physics, Peripheral neuropathies

## Abstract

Diabetic foot syndrome, a long term consequence of Diabetes Mellitus, is the most common cause of non-traumatic amputations. Around 8% of the world population suffers from diabetes, 15% of diabetic patients present a diabetic foot ulcer which leads to amputation in 2.5% of the cases. There is no objective method for the early diagnosis and prevention of the syndrome and its consequences. We test terahertz imaging, which is capable of mapping the cutaneous hydration, for the evaluation of the diabetic foot deterioration as an early diagnostic test as well as ulcers prevention and tracking tool. Furthermore, the analysis of our terahertz measurements combined with neurological and vascular assessment of the patients indicates that the dehydration is mainly related to the peripheral neuropathy without a significant vascular cause.

## Introduction

Diabetic foot syndrome is caused by the combination of vascular and neurological deterioration derived from Diabetes Mellitus^1^. These two conditions together produce loss of sensation and skin dehydration in the lower extremities which increase the probabilities to develop ulcers that, in many cases, lead to the amputation of the affected limb^2^. A diabetic foot ulcer occurs in approximately 15% of diabetic patients^3^, more than 50% of diabetic foot ulcers become infected^4^ and, 20% of diabetic foot infections result in amputation^5^. Unfortunately, a broadly accepted quantitative and objective test to early diagnose the risk of ulceration does not exist. The onset of diabetic peripheral neuropathy (DPN), which correlates with the formation of ulcers at later stages, is usually evaluated by the Semmes-Weinstein Monofilament (SWM) test^2^, which consists in poking the feet soles of the patient with a flexible tip, the patient should report when the pressure is felt. The test is considered positive after the patient misses a certain number of pokes^6^. In addition, the appearance of peripheral artery disease (PAD), which also correlates with the formation of ulcers, is diagnosed by the measurement of the Ankle-Brachial Index (ABI), which compares the blood flow in the arm and ankle arteries as an indicator of vascular deterioration^7^. However, these two techniques, which are used for the clinical evaluation of diabetic foot syndrome, are either subjective or indirect.

Terahertz (THz) radiation is extremely sensitive to the presence of water. This has lead to a number of applications in the biological^8,9^ and medical^10,11^ fields. Terahertz has been used to monitor the water content in biological tissues^12^, for the detection of skin and other types of cancer^13–17^, for the evaluation of skin burns^18,19^ as well as for understanding the interaction of dermatological products with skin^20^. Previously, we presented two proof-of-concept studies using terahertz imaging as a potential diagnostic technique for the diabetic foot syndrome^21,22^. The water content on the sole of the foot of a group of diabetics and non-diabetics was compared showing significant differences, yet, the studies could not be considered a clinical evaluation of the diagnostic test owing to the small number of subjects tested, a potential bias of age between a younger non-diabetic group and the diabetic group, and the lack of a careful application of golden standards for comparison.

In this article, we present a first evaluation of a technique which we call Moisture MApping by Terahertz (MMAT) as a diagnostic test of diabetic foot syndrome. The feet soles of non-diabetic and diabetic subjects were imaged with terahertz radiation. The presence of DPN and PAD were assessed by SWM and ABI, respectively, in a subgroup of the diabetic patients. A comparison between non-diabetic and diabetic subjects is presented as a normality study of the proposed technique. The diabetic patients were grouped and studied according to the complications related to the diabetic foot syndrome. The sensitivity, specificity and threshold values of the MMAT technique as diabetic foot diagnostic test are defined.

## Results

A total of 80 type-2 diabetic (45 female and 35 male) and 98 non-diabetic (70 female and 28 male) subjects were studied. We acknowledge the significant difference of the number of female and male subjects in the non-diabetic group, yet an analysis of the results shows that there is no significant difference between the female and male subgroups of the control group in terms of feet hydration (female 50.2±4.4% vol.; male 52.0±4.0% vol.). Diabetic subjects are diagnosed diabetes patients with clinically documented evolution of the disease recruited from the DiabetIMSS program at the Instituto Mexicano del Seguro Social (IMSS). The glucose levels of the group varied between 111 mg/dl and 149 mg/dl, while the HbA1c between 6.5% and 7.9%. Eight diabetic subjects had a history of foot ulcers, and six out of those had undergone partial lower limb amputation. The non-diabetic group was recruited among patients, with other unrelated conditions, and relatives from a waiting room at the Unidad Médica de Alta Especial (UMAE) IMSS-T1. All non-diabetic subjects underwent a “﻿quick” reactive-strip glucose test in order to verify the absence of diabetes. Of all the diabetic patients, 60 were tested with SMW and 59 with ABI. Both feet soles of all subjects were scanned with terahertz time-domain imaging as described in the “Methods” section and personal and clinical data relevant to the study were collected. The raw data from the terahertz images were analyzed as described in the “Methods” section in order to obtain the water content of the skin.

**Figure 1:**
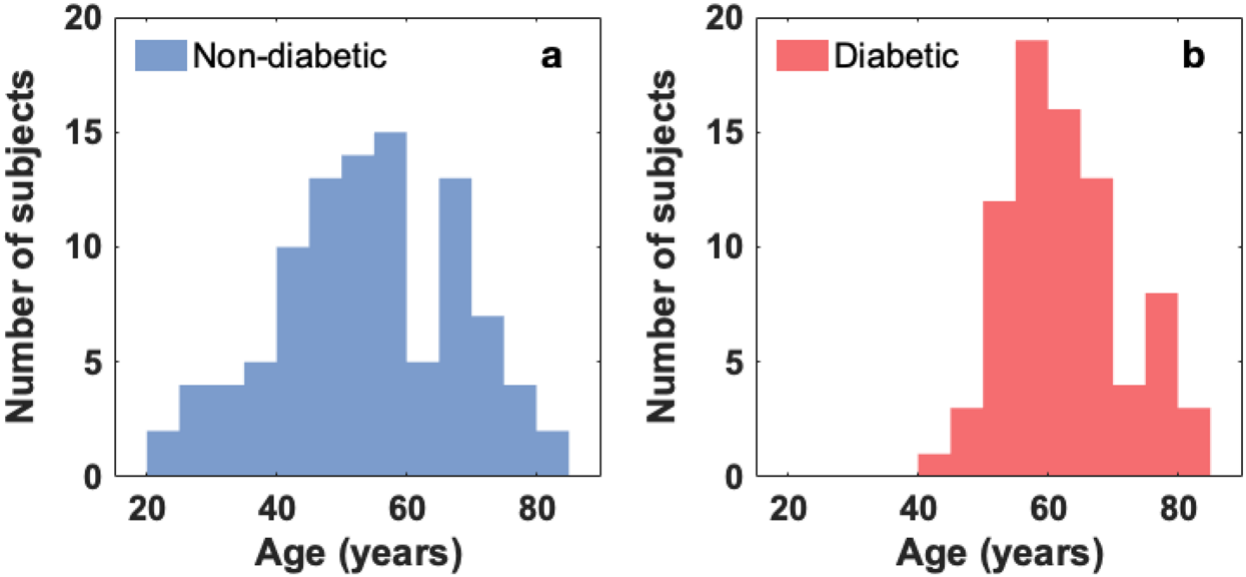
Age distribution of (**a**) non-diabetic group and (**b**) diabetic group.

### Hydration evolution with age

**Figure 2:**
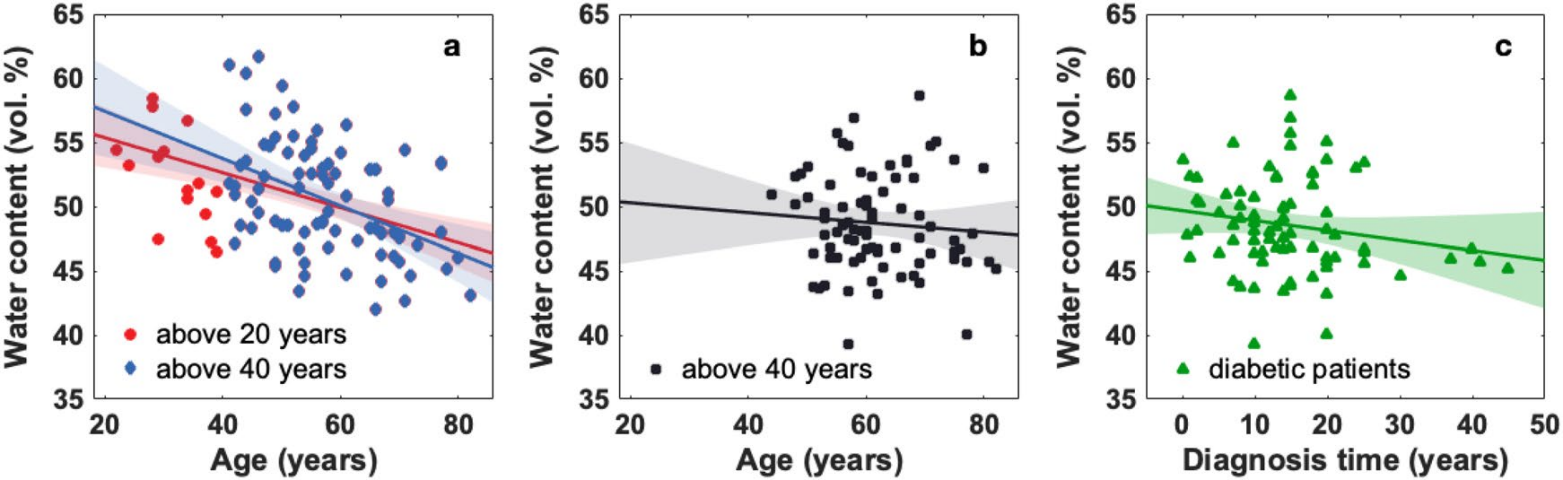
Average foot sole hydration evolution with age on (**a**) all non-diabetic subjects (red circles, $\mathrm {n}=98$) and non-diabetic subjects above 40 years old (blue diamonds, $\mathrm {n}=83$). (**b**) Diabetic subjects above 40 years old ($\mathrm {n}=80$). (**c**) Average foot sole hydration evolution as function of the time since diagnosis of diabetes. Linear fits to the data and confidence intervals are represented by solid lines and shaded areas, respectively.

It has been suggested that age might play an important role on the skin hydration^21^. In this study, the age of the non-diabetic subjects ranged from 20 to 82 years, whereas the diabetic patients were between 40 and 82 years old. The age distribution of both groups is shown in Figure 1. The evolution of the water content in the skin with age was analyzed. For the purpose of statistical analysis and in order to discard a bias owing to the difference in age distribution of the non-diabetic and diabetic groups, the non-diabetics were analyzed in two ways. Firstly the entire non-diabetic group and secondly only subjects older than 40 years of age which match more closely the age distribution of the diabetics. The relation between hydration and age is shown in Figure 2. Data points represent the hydration averaged over the foot sole for the foot of each subject. Linear fits to the data are represented by solid lines. The confidence intervals of the fits, which in turn denote the trend uncertainty, are represented by shaded areas. It is noticeable that young non-diabetic subjects present higher hydration values than older non-diabetic subjects while diabetic patients, all older than 40 years of age present a relatively flat evolution of their hydration as function of age. Yet, it is interesting to notice that there is a slight reduction of the hydration as a function of the time since patients were diagnosed with diabetes.

### Normality study: non-diabetic versus diabetic subjects

From the total of volunteers only 53 non-diabetic and 40 diabetic subjects in the age range from 40 to 60 years were considered for a normality comparison. This selection was done since we could only draw statistically significant conclusions in this age range owing to the small number of subjects on either of the groups outside this age interval. The water content averaged over the foot sole, at the center of the big toe and at the center of the heel was obtained, these two particular areas are more prone to generate ulcers in diabetic patients. The hydration values from both feet of each volunteer were averaged.

**Figure 3:**
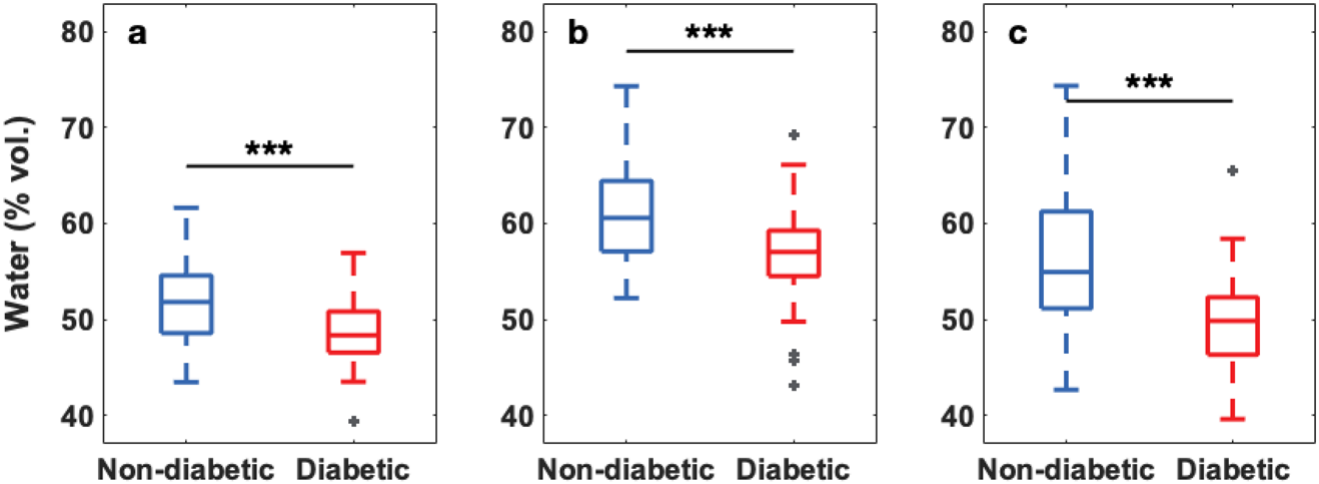
Skin hydration distribution (**a**) averaged over the foot sole ($\mathrm {p}=5.90\times 10^{-4}$), (**b**) at the center of the big toe ($\mathrm {p}=4.30\times 10^{-4}$) and (**c**) at the center of the heel ($\mathrm {p}=8.70\times 10^{-6}$) of non-diabetic ($\mathrm {n}=53,51,51$) and diabetic ($\mathrm {n}=40,31,38$) subjects, only subjects in the age range 40–60 years were considered since this is the region where there is significant population overlap in both groups, and either the toe or the heel of some subjects was excluded from the analysis for individual reasons such as previous amputation, local image occlusion owing to the presence of a bandage, etc. The box represents the middle 50% of the data, 25% of the data is below the lower bar, the middle bar represents the median and 75% of the data relies below the upper bar. The dark grey crosses indicate the outliers and the statistical significance is denoted by black stars displayed over the boxplots.

Figure 3 shows the hydration distribution (a) averaged over the foot sole, (b) at the center of the big toe and (c) at the center of the heel by subject group. We can see that, the hydration of the non-diabetic subjects are above the corresponding values for diabetics. The largest separation between groups is given by the hydration at the center of the heel (Fig. 3c).

### Classification of diabetic subjects

Diabetic patients were classified according to the complications associated to the diabetic foot syndrome. Since diabetic foot is defined as “a foot affected by ulceration that is associated with neuropathy and/or peripheral arterial disease of the lower limb in a patient with diabetes”^23^, diabetic patients with DPN and/or ulceration- and/or amputation- record, were considered as diabetics with complications, on the contrary, being diabetic patients without DPN nor ulceration nor amputations considered as diabetics with no complications. In other words, the existence of one or more of these three conditions was used as our golden standard. In order to evaluate DPN, the SWM test was performed on 60 diabetic patients. DPN was diagnosed in diabetic patients who scored less than 8 out of 10 SWM points.

**Figure 4:**
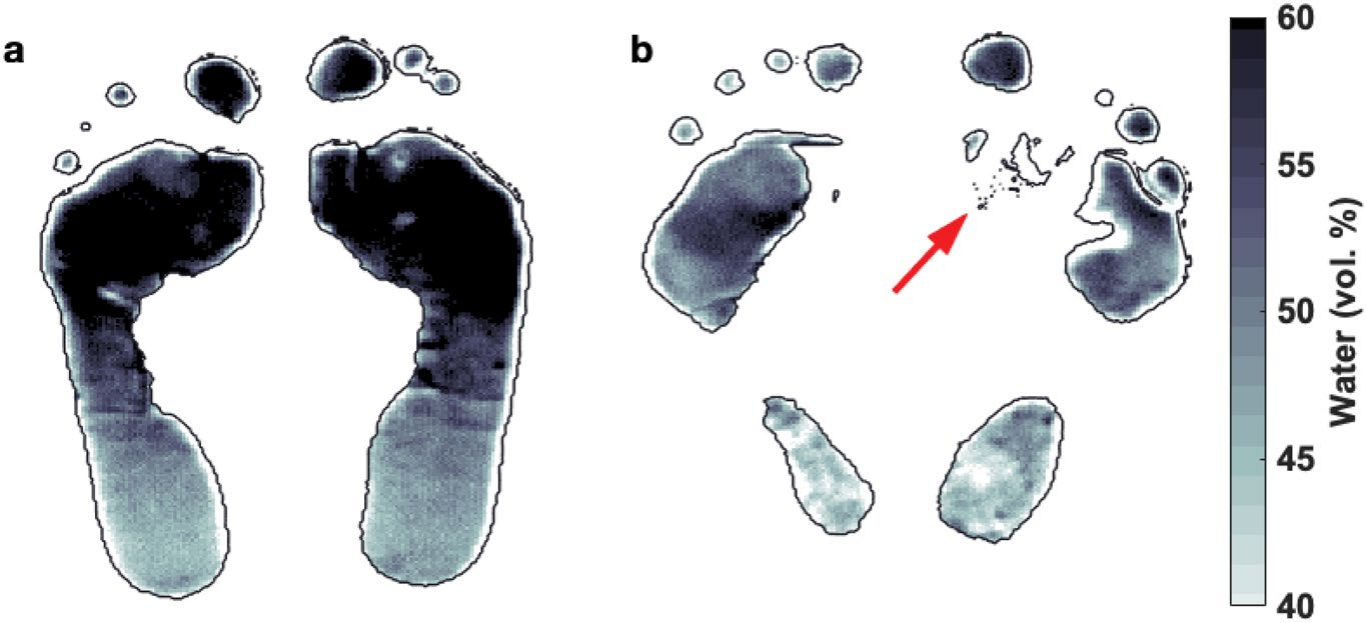
Hydration image of (**a**) a diabetic patient with no complications and (**b**) a diabetic patient with complications. An ulcer, covered by a patch (red arrow), is noticeable on the right metatarsal area of the diabetic patient with complications which, in turn, presents lower hydration along the foot sole compared to the diabetic patient with no complications.

Taking these test results and the ulcer/amputation records, from all the 80 diabetic patients, only 45 were considered in the “diabetics with no complications” group and 21 in the “diabetics with complications” group. In Figure 4, a terahertz hydration image of (a) a diabetic patient with no complications and of (b) a diabetic patient with complications can be observed. The terahertz hydration image in Fig. 4b corresponds to a diabetic patient with an ulcer in the right metatarsal area, indicated by a red arrow. During the terahertz scanning, the ulcer was covered by a patch, therefore the water content information was not obtained for this region.

The variation in the skin hydration is represented as a grey-scale color map ranging from 40% to 60%. For hydration levels below 40% and above 60%, the color map is fixed to white and black respectively, in order to highlight significantly dehydrated or hydrated skin regions.

**Figure 5:**
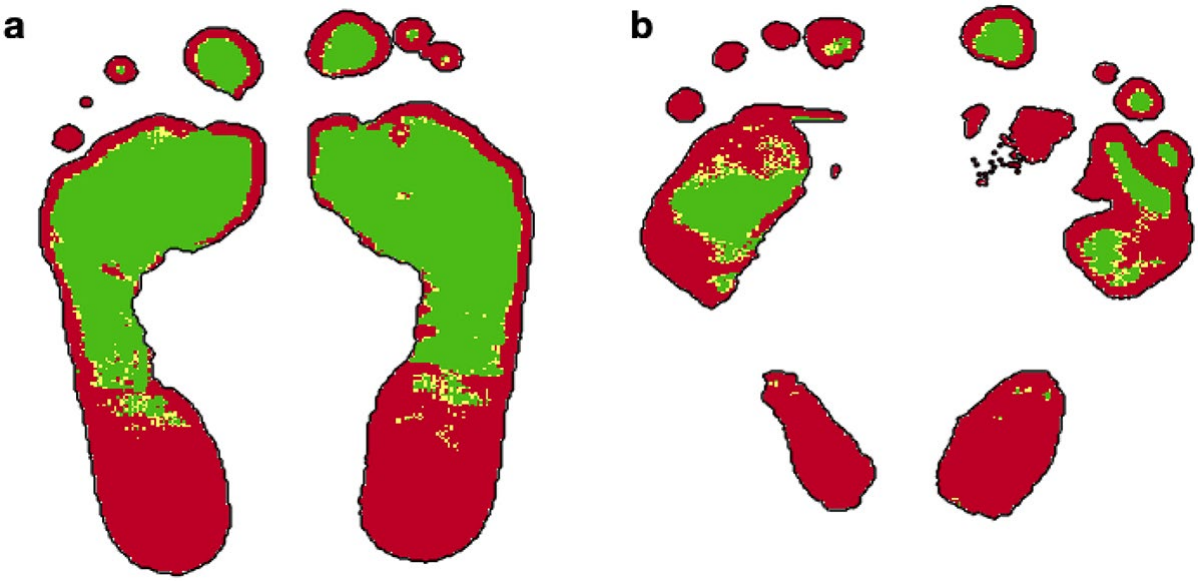
RYG images of (**a**) a diabetic with no complications and (**b**) a diabetic patient with complications. Red pixels, colored for hydration values below 51.7%, potentially indicate high deterioration risk. Green pixels, colored for hydration above 52.9%, are expected to have lower ulceration risk. Yellow pixels are colored for hydration values in between. Diabetic patients with complications present more red areas than the diabetics with no complications.

Terahertz hydration images give information of the water content pixel by pixel along the foot sole, but if the water content of the skin is color-coded and Red-Yellow-Green (RYG) images^22^ are formed, it is possible to easily visualize areas with low hydration, that could result in high risk of ulceration. Hydration thresholds were defined so pixels below $51.7\%$ were colored in red, pixels above $52.9\%$ were colored in green and pixels with values in between were colored in yellow. In Figure 5, RYG images of (a) a diabetic patient with no complications and (b) a diabetic patient with complications are shown as examples.

From Fig. 5, we can notice that even though the diabetic with no complications presents low hydration areas, they represent a significantly smaller fraction of the foot-sole area than those of the diabetic with complications feet. Furthermore, we can observe that the area surrounding the ulcer in the right metatarsal of the diabetic with complications, Fig. 5b, is highly deteriorated and so colored in red.

**Figure 6:**
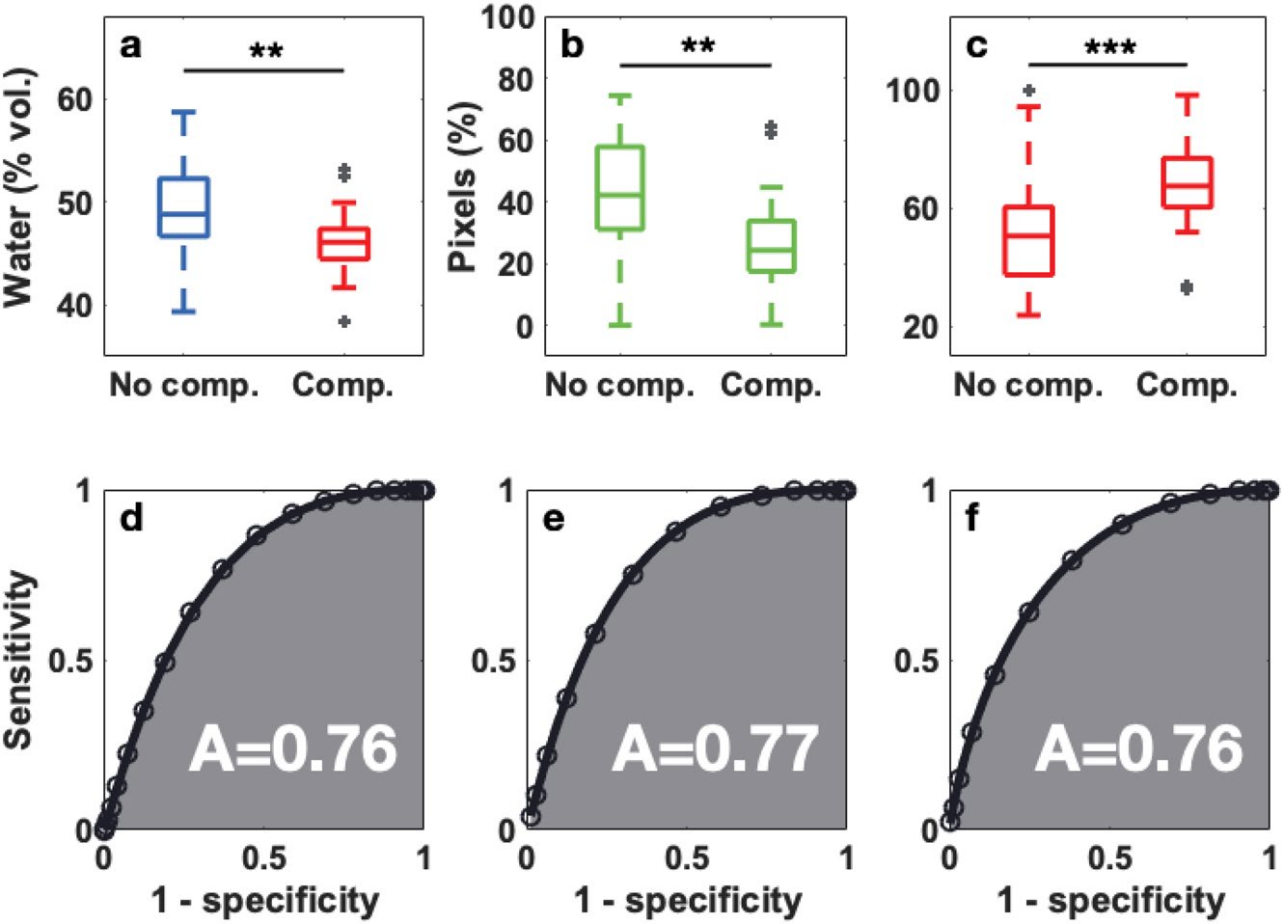
(**a**) Hydration averaged over the foot sole ($\mathrm {p}=0.0016$), (**b**) green pixels distribution ($\mathrm {p}=0.0011$) and (**c**) red pixels distribution ($\mathrm {p}=0.0010$) of diabetic patients with no complications ($\mathrm {n}=45$) and diabetic patients with complications ($\mathrm {n}=21$). Diabetic patients with complications present the less hydration and green pixels and the more red pixels which indicates higher skin deterioration. The (**d**–**f**) ROC curves represent the ability to discriminate between both groups according to (**a**–**c**), respectively.

The average hydration, and the green pixels and red pixels distribution over the foot sole of the diabetic subjects in both groups were obtained. The values of feet with ulcers and/or amputations were not considered, i.e., if a diabetic patient had had an ulcer or had already undergone an amputation on one of his/her feet, only the opposite-foot values were taken into account for this study. Then, considering that the higher the skin hydration the less the skin deterioration, Receiver-Operating Characteristic (ROC) curves, which represent a test potential to diagnose a disease^24,25^, were determined taking the average hydration, the percentage of green pixels and the percentage of red pixels as values for the diagnostic test. These distributions and the corresponding ROC curves can be observed in Figure 6. The larger the area under the ROC curve, the better the discrimination potential of the test.

From Fig. 6a we can see that the averaged hydration in the foot sole of diabetic patients with no complications is higher than of the diabetic patients with complications. Additionally, the fraction of green pixels (Fig. 6b) and red pixels (Fig. 6c) is larger and smaller, respectively, in diabetic patients with no complications than in diabetic patients with complications. The areas under the corresponding ROC curves, indicate that, the averaged hydration, and the green pixels and red pixels distribution values have the potential to classify diabetic patients according to the complications associated to the diabetic foot syndrome.

**Figure 7:**
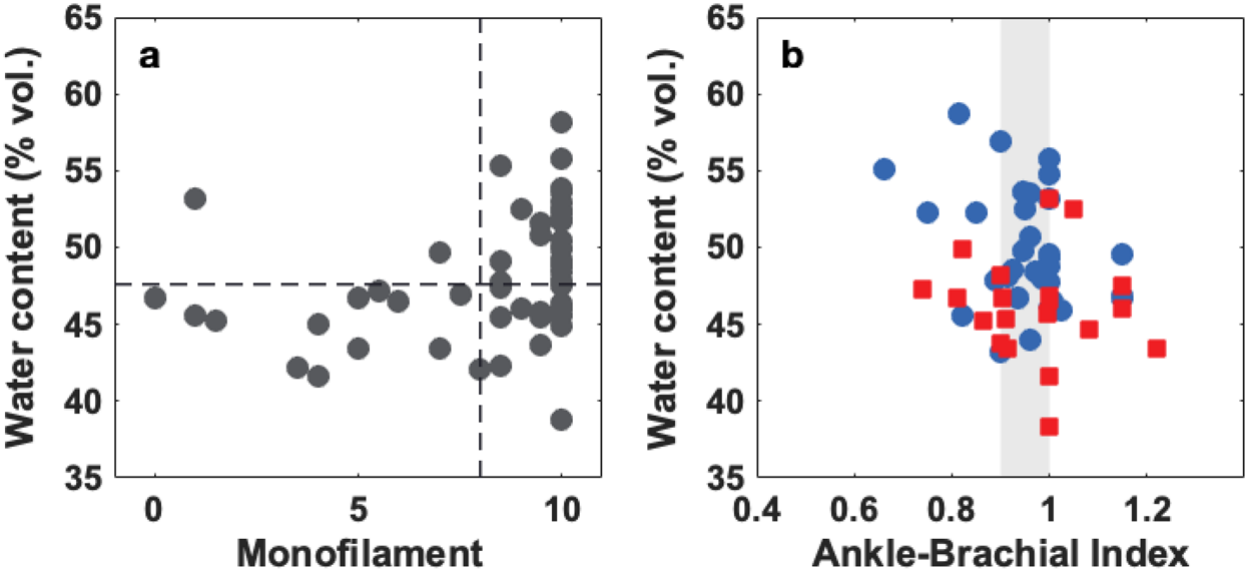
(**a**) Water content averaged over the two feet soles as a function of the minimum SMW score of the two feet for each patient ($\mathrm {n}=60$). (**b**) Water content averaged over the foot sole as a function of the ankle-brachial index (ABI) for diabetic patients with complications (red squares, $\mathrm {n}=22$) and diabetic patients with no complications (blue circles, $\mathrm {n}=32$). The grey shaded area indicates ABI “borderline” values (0.91–0.99), ABI values $\le$ 0.90 are considered abnormal, the ABI normal range is defined from 1.0 to 1.4.

In order to visualize the relation between the degree of neurological deterioration and the dehydration, Fig. 7a shows the water content averaged across the two feet of the patient as a function of the monofilament score, i.e. the number of correct pressure identification points out of ten. Two dashed lines are used to identify the hydration threshold of 47.6% and the 8/10 monofilament threshold. As seen in the plot, the great majority of the high-scored patients lie in the upper-right, while all except for two cases with scores below 8/10 lie in the lower-left quadrant. From this plot it is clear that there is a correlation between the presence of neuropathy and the reduction of hydration of the feet.

As previously mentioned, currently, diabetic foot is partly diagnosed evaluating PAD by ABI. In this study, 59 diabetic patients were tested with ABI. Subsequently, the ABI results were compared to the water content averaged over the feet soles of the diabetic patients, as shown in Figure 7b.

From Fig. 7b, we infer that there is no correlation between the vascular deterioration and the skin hydration in the foot in either the diabetic patients with complications (red squares) and the diabetic patients with no complications (blue circles). This suggests that the skin dehydration in diabetic patients is not related to a vascular deterioration but to a neurological one. The nervous system, particularly the sympathetic nervous system, regulates transpiration, therefore the evidence presented in this article suggests that the excessive dehydration of the feet-sole of diabetic patients is mainly caused by the feedback system that attempts to maintain a hydration homeostatic, rather than a physical reduction of the water flow carried by blood.

Finally, the sensitivity and specificity of the diagnostic test, as well as the threshold values for the diagnosis result were determined following the analysis described in the “Methods” section. The resulting MMAT test values are presented in Table 1.

**Table 1:** Sensitivity, specificity and threshold values of the MMAT diagnostic test regarding the average foot sole hydration and, the green pixels and red pixels distribution.

	Average	Green (%)	Red (%)
Sensitivity	81	76	76
Specificity	69	73	78
Threshold	47.6	32.5	61.5

Overall, the MMAT test for diagnosing diabetic foot shows that,

- The average water content over the foot sole diagnoses diabetic foot syndrome with a sensitivity of 81% and a specificity of 69%. The hydration threshold to be tested as positive or negative is 47.6%.
- The green pixels fraction diagnoses diabetic foot syndrome with a sensitivity of 76% and a specificity of 73% with a threshold value set to 32.5%.
- The red pixels fraction diagnoses diabetic foot syndrome with a sensitivity of 76% and a specificity of 78% with a threshold value set to 61.5%.

## Discussion

As part of the characterization of our populations we found that the hydration of non-diabetics has a clear decreasing dependence with age. Diabetics, on the other hand, show a lower hydration level, without a significant dependence on age. Yet, we do observe a slight decrease of hydration as a function of the time since diagnosis of diabetes. After excluding the age difference between the two populations, we found a moderate difference in the hydration of their feet soles, the big toe and the heel between the diabetics and the non-diabetics. Furthermore, significant hydration differences among diabetic subjects are observed when classified according to their ulcer/amputation history or the presence of neuropathy. In addition, we found that the presence of neuropathy, unlike the presence of vasculopathy, correlates with the reduction of hydration.

This study demonstrates a correlation between the complications related to the diabetic foot syndrome (DPN, ulcers and amputations) and the hydration in the skin of the foot sole. The red and green pixel statistics were also found to be strongly related to the deterioration in the foot of diabetic patients. Moreover, it was found that there is no correlation between ABI values and hydration of the skin suggesting that the reduced dehydration observed in diabetic patients is caused by the impossibility of the sympathetic nervous system to maintain the hydration levels, rather to a vascular problem. Yet, the quantitative analysis together with the terahertz images presented in this study propose that the MMAT technique has potential as an early diagnostic test for diabetic foot syndrome by evaluating the skin hydration and relating it to the deterioration on the foot sole. Terahertz hydration images together with RYG images may help in the diagnosis, treatment and evaluation of the disease. The sensitivity and specificity values estimated in the range from 76% to 81% and 69% to 78%, respectively, are encouraging for a non-invasive, objective and direct diabetic foot diagnostic test.

An important aspect of this study is that it significantly improves our previously reported findings^21^. The current contribution contains a much larger sample with better age match between the groups and better assessment of the state of the feet of the diabetic subjects. In addition, the control group was properly screened for the absence of diabetes.

## Methods

### Terahertz Time Domain Imaging (THz-TDI)

**Figure 8:**
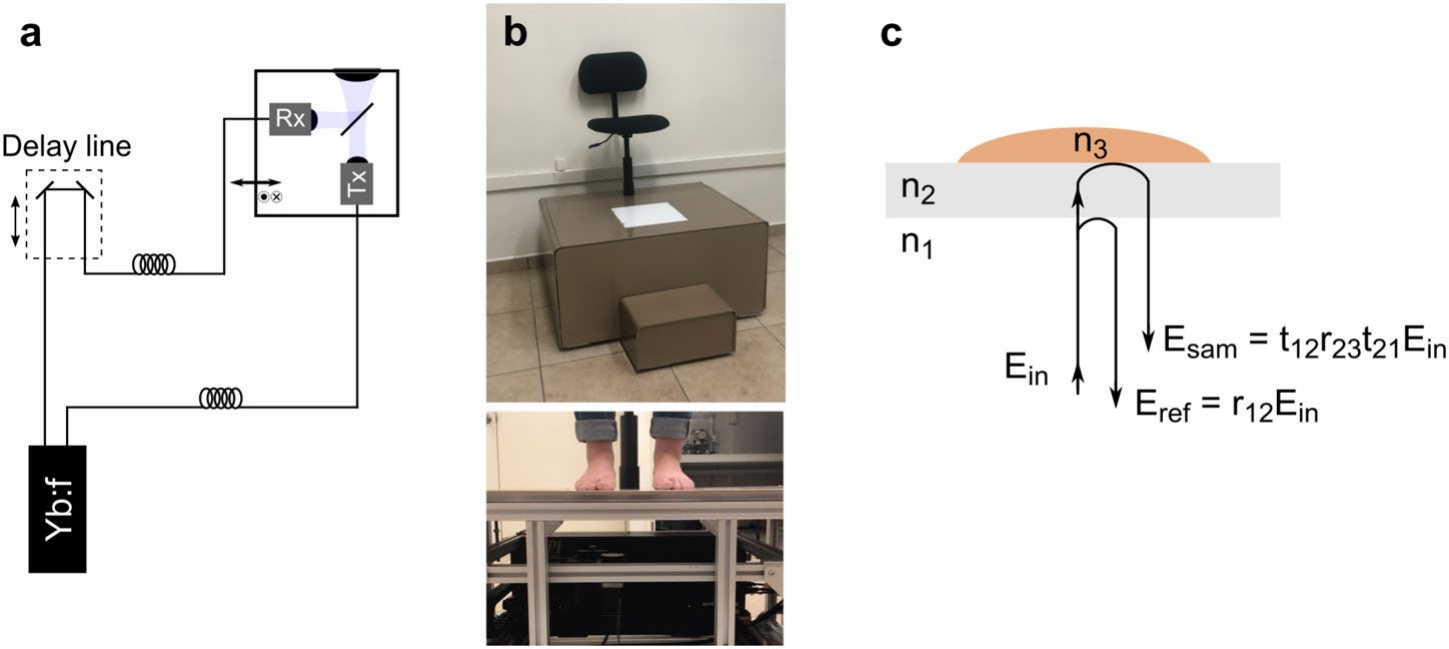
(**a**) Schematic representation of the fiber-coupled THz-TDS system used. (**b**) Photographs of the MMAT platform built for the acquisition of the THz images of the feet of patients. (**c**) Ray schematic of the terahertz radiation path.

Terahertz images were acquired using an Advanced Photonix, Inc. (API) TeraGauge spectrometer coupled to an API Imaging Platform. The TDS system, schematically shown in Fig. 8a, is based on a Yb:Fiber pulsed laser. The femtosecond pulses are split into two parts, the first one is sent to a photoconductive transmitter to produce terahertz radiation, while the second one is sent to a delay-line and then to a photoconductive receiver in order to trigger the terahertz detection. The transmitter and receiver are coupled with the AXA5001 Collinear Adapter in order to form a normal-incidence transceiver for reflection geometry. The collinear adapter is mounted on the imaging platform which consists of rails and motion controllers to perform raster scans.

### Imaging of subjects’ feet

The Moisture MApping by Terahertz (MMAT) scanner, shown in Fig 8b, consists of an elevated platform with a high-density polyethylene window, where the subject’s feet are placed. The terahertz imaging system is placed below the measurement window to scan both feet soles. Each terahertz image consists of a collection of terahertz waveforms taken across a 240 x 245 points area spaced by 1 mm.

All diabetic patients were requested not to apply any moisturizing products before the image acquisition. Non-diabetic subjects were chosen randomly, information about the application of moisturizing products on the feet was collected. The measurement window was cleaned up with isopropanol before both feet were placed for the image acquisition. The scanning time was less than 10 min per terahertz image.

### Water content retrieval analysis

In order to obtain the water content in each pixel of the terahertz image, the reflected terahertz radiation is analyzed by comparing the experimental transfer function with the analogous theoretically modeled transfer function, as described in^21^.

A terahertz pulse, $\hbox {E}_{\mathrm{in}}$, incides into the measurement polyethylene window, as observed in Fig. 8c. A part of the pulse is reflected at the surface and is given by

1 $$\begin{aligned} \hbox {E}_{\mathrm{ref}} = \hbox {r}_{12}\hbox {E}_{\mathrm{in}}. \end{aligned}$$

The transmitted part of the pulse propagates through the polyethylene window, is partly reflected at the window-sample interface, transmitted back through the window to finally be partly transmitted at the window-air interface and given by

2 $$\begin{aligned} \hbox {E}_{\mathrm{sam}} = \hbox {t}_{12}\hbox {r}_{23}\hbox {t}_{21}\hbox {E}_{\mathrm{in}}. \end{aligned}$$

The $\hbox {t}_{\mathrm{ij}}$ and $\hbox {r}_{\mathrm{ij}}$ expressions are the transmission and reflection Fresnel coefficients between the *i*-th and *j*-th materials’ interface. The reflected waveform measured as a function of time consists of two pulses, $\hbox {E}_{\mathrm{ref}}$ and $\hbox {E}_{\mathrm{sam}}$. The spectral amplitude of these two pulses defines the transfer function of the terahertz radiation, given by

3 $$\begin{aligned} \hbox {H}_{\mathrm{theo}}(\omega ) = \frac{\left| \hbox {E}_{\mathrm{sam}}(\omega ,\eta )\right| }{\left| \hbox {E}_{\mathrm{ref}}(\omega )\right| } = \left| \frac{\hbox {t}_{12}(\omega )\hbox {r}_{23}(\omega ,\eta )\hbox {t}_{21}(\omega )}{\hbox {r}_{12}(\omega )}\right| , \end{aligned}$$

where the Fresnel coefficients are calculated from the refractive indices of the materials. The refractive indices of air and high-density polyethylene are constant and well known, the refractive index of the sample depends on the water content of the skin. From the experimental measurements, the pulses separated in time are Fourier transformed to build the experimental transfer function

4 $$\begin{aligned} \hbox {H}_{\mathrm{exp}}(\omega )=\frac{\left| \hbox {E}_{\mathrm{sam}}(\omega )\right| }{\left| \hbox {E}_{\mathrm{ref}}(\omega )\right| }. \end{aligned}$$

An empirical calibration factor^26^ must be introduced in order to compensate for the slight misalignment of the two beams. This calibration factor is obtained with no sample placed on the measurement window and the reflected pulses are associated to the air-polyethylene and polyethylene-air interfaces. The refractive indices of both, air and polyethylene, materials are well know, therefore, the calibration factor is calculated as

5 $$\begin{aligned} \hbox {A}_{\mathrm{cal}}(\omega ) = \frac{\left| \hbox {E}_{\mathrm{ref}}(\omega )\right| }{ \left| \hbox {E}_{\mathrm{air}}(\omega )\right| }\left| \frac{\hbox {r}_{12}(\omega )}{\hbox {r}_{23,\mathrm{air}}(\omega )\hbox {t}_{12}(\omega )\hbox {t}_{21}(\omega )}\right| , \end{aligned}$$

and incorporated to the theoretical transfer function, Eq. (3), to be redefined by

6 $$\begin{aligned} \hbox {H}_{\mathrm{theo}}(\omega ) = \left| \frac{\hbox {t}_{12}(\omega )\hbox {r}_{23}(\omega ,\eta )\hbox {t}_{21}(\omega )}{\hbox {r}_{12}(\omega )}\right| \hbox {A}_{\mathrm{cal}}(\omega ). \end{aligned}$$

The Fresnel coefficients in this expression only depend on the refractive index of polyethylene and air, except for $\hbox {r}_{23}(\omega ,\eta )$, which, for normal incidence, is given by

7 $$\begin{aligned} \hbox {r}_{23}(\omega ,\eta )=\frac{\hbox {n}_{\mathrm{HDPE}}-{{\tilde{\hbox {n}}}}_{\mathrm{skin}}(\omega ,\eta )}{\hbox {n}_{\mathrm{HDPE}}+{{\tilde{\hbox {n}}}}_{\mathrm{skin}}(\omega ,\eta )} \end{aligned}$$

with

8 $$\begin{aligned} {{{\tilde{\hbox {n}}}}_{\mathrm{skin}}(\omega ,\eta )=\sqrt{\varepsilon _{\mathrm{mix}}(\omega ,\eta )}} \end{aligned}$$

where ${\varepsilon _{\mathrm{mix}}(\omega ,\eta )}$ is the dielectric function of human skin, given by

9 $$\begin{aligned} \root 3 \of {\varepsilon _{\mathrm{mix}}(\omega ,\eta )}=\eta \root 3 \of {\varepsilon _{\mathrm{water}}(\omega )}+(1-\eta )\root 3 \of {\varepsilon _{\mathrm{dry}\,\mathrm{skin}}(\omega )}, \end{aligned}$$

determined by the LLL model of effective medium theory^27^, considered as a combination of water and dry skin, where the dielectric function of water, $\varepsilon _{\mathrm{water}}$, is well known for terahertz frequencies^28^ and the dielectic function of dry skin, $\varepsilon _{\mathrm{dry}\,\mathrm{skin}}$, was determined as explained in^21^. Finally, the water content at each measured point was obtained by minimizing the difference between the experimental and theoretical transfer functions by varying the parameter $\eta$ in a least square fitting algorithm.

### Analysis of statistical significance

We performed a significance analysis in order to determine if the differences observed between the groups in the Normality Study and the Classification of Diabetic Subjects sections we concluded were statistically significant (*p* values ranged in the $8\times 10^{-6}$ and $1.6\times 10^{-3}$ for all cases as indicated in the relevant plots). The data statistical significance was determined performing the one-way analysis of variance (ANOVA) using the Statistics Toolbox of MATLAB. The p-value was calculated with a 5% threshold for each data set.

### Analysis of the diagnostic test accuracy

In order to evaluate the capability of the MMAT diagnostic test to accurately classify subjects into diseased or non-diseased, a Receiver-Operating Characteristic (ROC) analysis was performed as detailed in^24,25^. 2x2 contingency tables were built following the Table 2.

**Table 2:** 2x2 contingency table. Diagnostic test result in relation to the golden standard result. Subjects with diagnostic test values less than the threshold are classified as positive and subjects with diagnostic test values greater than or equal to the threshold are classified as negative.

Diagnostic test result	Golden standard result	
	Diseased	Non-diseased
Positive	True positives	False positives
Negative	False negatives	True negatives

For the classification of the diabetic subjects, an SWM test lower or equal to 8 out of 10 and/or a history of ulcer or amputation were considered as golden standards. The threshold value for the diagnostic test result was defined in terms of water content in the skin and the red/green pixels distribution over the foot sole.

By varying the water content threshold, sensitivity, $\hbox {sens.} = \hbox {TP}/(\hbox {FN}+\hbox {TP})$, and specificity, $\hbox {spec.} = \hbox {TN}/(\hbox {FP} + \hbox {TN})$, values were calculated and the ROC curve was plotted. The area under the curve was determined. The optimal threshold value was found by minimizing the Euclidean index^29^, $\hbox {d}^2=(1-\hbox {sens.})^2+(1-\hbox {spec.})^2$, and then by optimizing the resulting threshold value for a higher sensitivity value.

### Ankle-brachial index test

In order to determine the ankle-brachial index (ABI), an Aloka branded Doppler ultrasound equipment with a 7.5 MHz linear transducer was used. For the measurement, the patient lies in a supine position for 10 minutes, the brachial systolic blood pressure (SBP) is measured for both arms and the highest value is recorded. Subsequently, with a cuff of width between 10-12 cm, the SBP of the anterior tibial and pedis arteries of both limbs. The highest value is recorded. The ABI is then calculated from the relationship between both pressures taken and selected, dividing the SBP of the lower limb by that of the upper limb. For subjects with no peripheral arterial disease, the SBP of both legs should be equal to or greater than that of the upper limbs by approximately 10-15 mmHg, owing to a larger peripheral resistance of the legs. The formula to be used for each limb consists of obtaining the ratio between the highest systolic pressure obtained in an ankle, with respect to the highest value brachial systolic pressure^30^.

### Semmes–Weinstein monofilament evaluation

**Figure 9:**
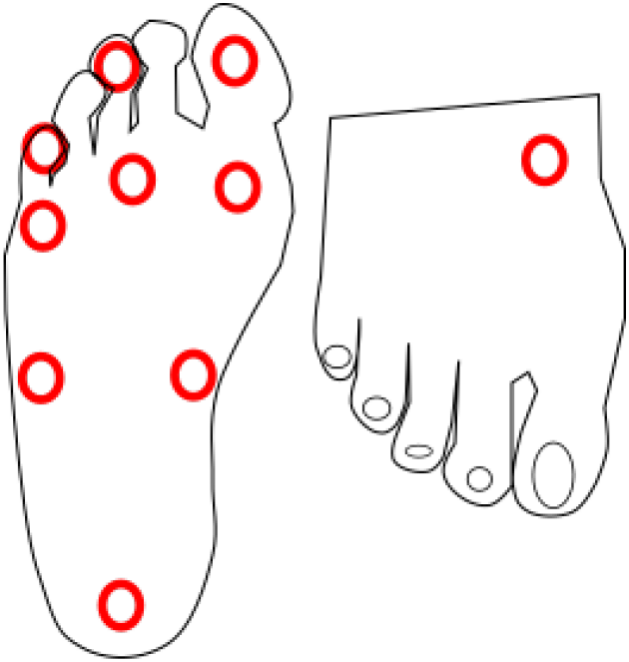
Schematic representation of the 10-point pattern used for the monofilament neuropathy evaluation.

The SWM^31^ was performed by using a 10 g monofilament on 10 sites on each foot. Patients were positioned laying and facing up without visual access to their own feet. The plantar surfaces of the first (NO. 1), third (NO. 2) and fifth digits (NO. 3); the plantar surfaces of the first (NO. 4), third (NO. 5) and fifth metatarsal heads (NO. 6); two positions side-by-side at the plantar medial region of the mid-foot (NO. 7 and 8); the plantar area of the heel (NO. 9) and the dorsal medial side of the mid-foot (NO. 10) as shown in Fig. 9 were probed with the monofilament.

The following procedure was used. Patients responded affirmatively each time they felt the application of the monofilament. Based on the outcome of the 10-point evaluation, each foot was classified independently. Yet, if a patient did not perceive the monofilament on more than two positions for either foot, the SWM was classified as abnormal, and the patient was accounted as showing deterioration due to peripheral neuropathy.

### Ethics commitee authorization

This is an observational study of cross section of screening of the feet of diabetic patients, performed at the medical unit of high specialty number 1 (Delegacion Guanajuato) of the Instituto Mexicano del Seguro Social.

This protocol was approved by the Institutional Review Board (IRB) National Commission for Scientific Research of the Instituto Mexicano del Seguro Social (R-2018-785-031, ammendments FE-2020-785-17 and FE-2020-785-18). Before taking part in the study, all volunteers (diabetics and non-diabetics) were informed about the objectives and implications of the study and signed an informed consent form.

All methods were carried out in accordance with relevant guidelines and regulations.

## Data Availability

The data that support the findings of this study are available from the corresponding author upon reasonable request. The software to process the terahertz data that supports the findings of this study is available from the corresponding author upon reasonable request.
